# Xanthogranuloma of the intrasellar region presenting in pituitary dysfunction: a case report

**DOI:** 10.1186/1752-1947-6-119

**Published:** 2012-04-29

**Authors:** Takamasa Nishiuchi, Koji Murao, Hitomi Imachi, Yoshio Kushida, Reiji Haba, Nobuyuki Kawai, Takashi Tamiya, Toshihiko Ishida

**Affiliations:** 1 Division of Hematology, Endocrinology and Metabolism, Department of Internal Medicine, Faculty of Medicine, Kagawa University, 1750-1, Miki-cho, Kita-gun, Kagawa, 761-0793, Japan; 2 Department of Diagnostic Pathology, Faculty of Medicine, Kagawa University, Kita-gun 761-0793, Kagawa, Japan; 3 Department of Neurological Surgery, Faculty of Medicine, Kagawa University, Kagawa University, Kita-gun 761-0793, Kagawa, Japan

**Keywords:** xanthogranuloma, intrasellar region, craniopharyngioma, Rathke's cleft cyst, panhypopituitarism

## Abstract

**Introduction:**

Differentiation of cystic mass lesions of the sellar and parasellar regions may pose a diagnostic dilemma for physicians, neurosurgeons, radiologists and pathologists involved in treating patients with these entities. A considerable number of tumors previously identified as craniopharyngiomas may, in fact, have been xanthogranulomas. We report a case of pituitary dysfunction caused by xanthogranuloma of the intrasellar region.

**Case presentation:**

A 47-year-old man of Japanese descent presented to our institution with a tumor located exclusively in the intrasellar region which manifested as severe hypopituitarism. MRI revealed a clearly defined intrasellar mass that was heterogeneously hyperintense on T1-weighted images and markedly hypointense on T2-weighted images. We preoperatively diagnosed the patient with Rathke's cleft cyst or non-functioning pituitary adenoma. Although the tumor was completely removed using a transsphenoidal approach, the improvement of the patient's endocrine function was marginal, and continued endocrine replacement therapy was needed. Postoperatively, a histological examination revealed the tumor to be a xanthogranuloma of the intrasellar region. His visual field defects and headache improved.

**Conclusion:**

Because diagnosis depends on surgical intervention and xanthogranulomas of the intrasellar region are very rare, the natural history of xanthogranuloma is still unknown. Therefore, this entity is difficult to diagnose preoperatively. We suggest that xanthogranuloma should be included in the differential diagnosis, even in the case of sellar lesions, to formulate appropriate postoperative management and improve endocrine outcomes.

## Introduction

Intracranial xanthogranulomas arise most commonly in the choroid plexus, almost uniformly in the trigone of the lateral ventricle, whereas xanthogranulomas of the sellar region are quite rare [1]. In some cases, a significant degree of overlap in these features occurs, and many xanthogranulomas of the sellar region may have been incorrectly identified as craniopharyngiomas. One study found that 37 craniopharyngiomas (33.6%) consisted predominantly of a xanthogranulomatous component [2]. Xanthogranulomas, also known as cholesterol granulomas, are granulomatous lesions characterized by cholesterol clefts, hemosiderin deposits, multi-nucleated foreign body giant cells, foamy macrophage accumulation and fibrous proliferation [2-6]. It is not clear, however, whether these additional characteristics represent a distinct entity, given the greater number of characteristics that xanthogranulomas share with craniopharyngiomas. Because the diagnosis depends on surgical intervention, the natural history of xanthogranulomas is unknown. Therefore, it is difficult to diagnose them preoperatively. Furthermore, when xanthogranuloma components predominate in the lesion, differential diagnosis of various lesions with xanthogranuloma is difficult [7,8]. Additionally, no typical radiological signs exist for xanthogranulomas [9]. Differentiation of cystic mass lesions of the sellar and parasellar regions may pose a diagnostic dilemma for neurosurgeons, radiologists and pathologists involved in treating patients with these entities. Establishing an accurate working diagnosis for sellar region pathology or histology is critical in predicting the likelihood of lesion recurrence and guiding postoperative adjunctive management. Therefore, further reports are required so that clinicians can gain greater insight into the clinical course, management and associated outcome of xanthogranuloma. Herein we report the case of a patient with pituitary dysfunction caused by xanthogranuloma of the intrasellar region.

## Case presentation

A 47-year-old man of Japanese origin presented with a history of headache, general fatigue and appetite loss of 3 months' duration. He was 178.2 cm in height and weighed 57 kg. His blood pressure was 90/50 mmHg, and his pulse was 64 beats/minute. His clinical examination showed the patient to be alert with initial signs of upper-visual-field defects. His physical examination revealed no absence of axillary or pubic hair and no neurological abnormalities. The results of the initial laboratory examinations conducted to determine hormone status are presented in Table 1. The anterior pituitary provocation test with corticotropin-releasing hormone, growth hormone-releasing hormone, gonadotropin-releasing hormone and thyrotropin-releasing hormone revealed impairment or low response of the secretory functions of cortisol, adrenocorticotropin, growth hormone, follicle-stimulating hormone, luteinizing hormone, thyroid-stimulating hormone and prolactin (Table 2). These abnormal responses to hormone stimulation, along with the patient's hormonal status, were indicative of hypopituitarism, specifically, hypogonadotropic hypogonadism.

**Table 1:** Laboratory data of the patient^a^

Parameter	Laboratory data	Normal range
ACTH (pg/ml)	5	4 to 48
Prolactin (ng/ml)	1.0	0 to 10
GH (ng/ml)	0.3	0 to 5
IGF-I (ng/ml)	30	264 to 542
TSH (μIU/ml)	2.013	0.3 to 5.0
ADH (pg/ml)	0.3	0.3 to 4.2
LH (mIU/ml)	0.9	> 0.5
FSH (mIU/ml)	2.7	> 1.0
Testosterone (ng/dl)	< 70	330 to 740
Cortisol (μg/dl)	2.2	5 to 17.9
F-T4 (ng/dl)	0.56	0.97 to 1.69
White blood cells (cells/μl)	5700	4700 to 8700
Red blood cells (×10^4^/μl)	375	400 to 540
Hemoglobin (g/dl)	11.1	13 to 17
LDH (U/l)	236	90 to 280
GOT (U/l)	65	8 to 40
GPT (U/L)	81	4 to 40
Na^+ ^(mEq/L)	142	135 to 145
K^+ ^(mEq/L)	4.4	3.5 to 5.0
Cl (mEq/L)	103	98 to 108
Ca^2+ ^(mg/dl)	8.4	8.5 to 10.0
Glucose (mg/dl)	74	65 to 110
Plasma osmolality (mOsm/kg H_2_O)	287	284 to 294
Urine osmolality (mOsm/kg H_2_O)	446	200 to 900
Urine 17-OHCS (mg/day)	0.6	3 to 9
Urine 17-KS (mg/day)	3.5	3 to 11

^a^Laboratory data are from initial evaluation. ACTH, adrenocorticotropin; GH, growth hormone; IGF-I, insulin-like growth factor I; TSH, thyroid-stimulating hormone; ADH,; LH, luteinizing hormone; FSH, follicle-stimulating hormone; F-T4, free thyroxine; LDH, lactate dehydrogenase; GOT, glutamic oxaloacetic transaminase; GPT, glutamic pyruvic transaminase; 17-OHCS, 17-hyroxycorticosteroid; 17-KS, 17-ketosteroid.

**Table 2:** Results of the anterior pituitary provocation test; adrenocorticotropic hormone and cortisol response for corticotropin-releasing hormone stimulation, growth hormone response for growth hormone-releasing hormone stimulation, luteinizing hormone and follicle-stimulating hormone response for gonadotropin-releasing hormone stimulation^a^

Parameter	0 minutes	30 minutes	60 minutes	90 minutes
TSH (μIU/ml)	2.592	5.357	5.206	4.564
PRL (ng/ml)	1	1	1	1
ACTH (pg/ml)	12	17	21	18
Cortisol (μg/dl)	4.7	6.3	8	5.4
GH (ng/ml)	0.1	1	1.2	0.9
LH (mIU/L)	0.9	3	3.4	3.2
FSH (mIU/L)	2.7	2.9	3.1	3.6

^a^TSH: thyroid-stimulating hormone, PRL: prolactin, ACTH: adrenocorticotropic hormone, GH: growth hormone, LH: luteinizing hormone, FSH: follicle-stimulating hormone.

**Figure 1:**
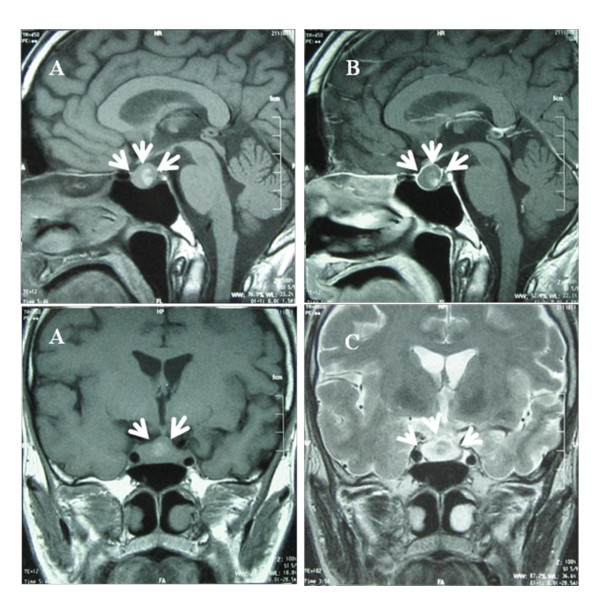
**MRI scan demonstrating a clearly defined intrasellar mass**. **(A) **and **(B) **Preoperative sagittal T1-weighted MRI (T1WI) showing a suprasellar round tumor measuring 15 × 20 mm in diameter (arrows) (A) and heterogeneous enhancement by gadolinium (Gd) contrast medium (T1WI+Gd) (arrows) (B). **(C) **Coronal T2-weighted MRI (T2WI) showing the tumor as partially hypointense (arrows).

MRI showed a clearly defined, intra- and suprasellar lesions with a diameter of 12 × 18 × 15 mm. The lesion appeared as mainly isointense to gray matter with some hyperintense areas and showed inhomogeneous contrast enhancement in the T1-weighted images (Figures 1A and 1B). The mass was also heterogeneously iso- or hypointense on T2-weighted images (Figure 1C).

**Figure 2:**
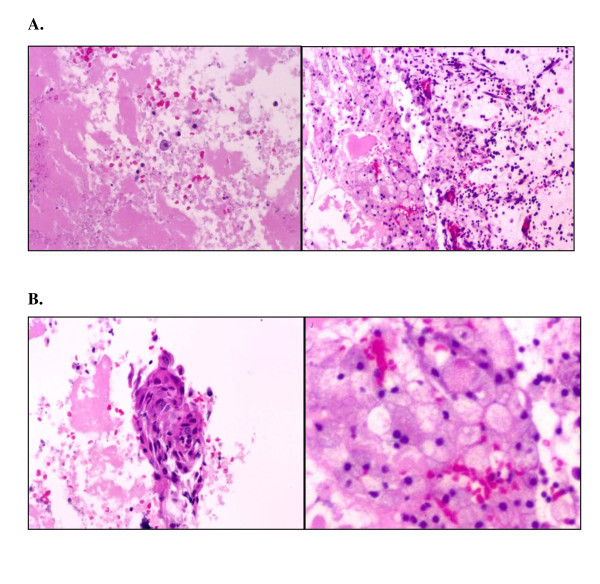
**Photomicrographs of the resected tumor showing that the tumor consisted of lymphoid or foamy macrophage infiltration (A) and (B) with small clusters of epithelial cells**. **(A) **Original magnification, ×200 (hematoxylin and eosin stain (H & E)). **(B) **Original magnification, ×400 (H & E).

On the basis of the above-mentioned examinations, our preoperative diagnosis was Rathke's cleft cyst (RCC) or non-functioning pituitary adenoma associated with hypophysitis. The lesion was completely removed by surgical exploration via transsphenoidal selective tumorectomy 5 months after clinical onset. Our pathological examination of the pituitary tumor revealed that the tumor was composed of abundant small epithelial cells with foamy macrophage or lymphoid infiltration, some of which were undergoing necrosis (Figure 2A). Hemosiderin deposits were also observed (Figure 2B). Atypically, there was no evidence of calcification or remarkable cholesterol clefts. On the basis of the histopathological examination, we confirmed the presence of an intrasellar xanthogranuloma and no observed cholesterol clefts.

Postoperatively, the patient's visual field defects and headache improved. However, he needed to continue endocrine replacement therapy (20 mg/day of hydrocortisone and 25 to 50 μg/day of thyroxine). MRI showed that the mass had been completely removed and revealed no evidence of tumor remnants.

## Discussion

The spectrum of cystic pathology occurring in the sellar region includes craniopharyngiomas, RCCs, colloid cysts, arachnoid cysts, cystic pituitary adenomas, epidermoid cysts, dermoid cysts and xanthogranulomas [10,11]. Xanthogranulomas have been reported to occur at various sites inside and outside the cranial vault [2]. However, xanthogranuloma of the sellar region is very rare. Xanthogranuloma of the sellar region was first reported in 1988 [3], with only 4 of 211 sellar and juxtasellar tumors (1.9%) showing the features typical of cholesterol granulomas. Paulus *et al. *described and classified xanthogranuloma of the sellar region as a distinct entity, after which the World Health Organization accepted that xanthogranuloma of the sellar region differs from classical craniopharyngioma in several ways: age at onset, anatomical site and localization of the lesion, and symptoms and prognosis [2,12].

The histogenesis and pathogenesis of xanthogranuloma remain unclear, and these details are controversial and probably heterogeneous. It is generally thought that the xanthogranulomatous reaction is induced mainly by chronic inflammatory processes in circumscribed areas of craniopharyngiomas or at the cyst wall and adjacent tissue of RCCs [2,13]. Degenerative changes in pituitary adenomas also may act as triggers [3], and extreme adeno- or infundibuloneurohypophysitis may result in giant cell granuloma. Secondary hypophysitis can arise from bacterial, viral and fungal infections, often in an immunocompromised host, or from systemic disorders such as autoimmune diseases, Langerhans histiocytosis and Erdheim-Chester disease. On the other hand, Paulus *et al. *[2] proposed xanthogranuloma of the sellar region as a distinct entity and found hemosiderin deposits in almost all (97%) of their 37 cases as a result of obstruction of a cavity, hemorrhage or both. Compared to classical craniopharyngiomas, xanthogranulomas characteristically occur in adolescents and young adults, predominantly at an intrasellar location, and are smaller in size and concomitant with more severe endocrine deficiencies, but they are more easily resected and lead to good treatment outcomes. Craniopharyngiomas usually arise in the pituitary stalk and grow, at least during the initial stage, mainly in the suprasellar space. The tumor in our patient was found at a classical location for RCCs, pituitary adenomas or hypophysitis, that is, the intrasellar portion and the sella turcica [2,5,6,9], although the adenomatous lesion was not included histologically.

Typical features of a xanthogranuloma of the sellar region include cholesterol clefts, lymphoplasmacellular infiltrates, marked hemosiderin deposits, multi-nucleated foreign body giant cells around the cholesterol clefts, accumulation of macrophages and only small epithelial cell clusters [2]. These features have been found in almost all cases of sellar xanthogranuloma. It is not clear, however, whether the additional characteristics represent a distinct entity, given the greater number of common characteristics. Indeed, in one study, 8% of xanthogranulomas of the sellar region had histological features consistent with adenomatous craniopharyngioma, whereas 35% (13 of 37) had squamous or columnar epithelium, but no other evidence of RCCs [2]. On the basis of these considerations and the histological findings in our patient, we speculate that our patient initially may have had RCCs or hypophysitis.

For xanthogranulomas, no typical radiological signs exist [9]. Although it is not always possible to identify the component of each signal intensity during surgery, researchers speculated in a previous report that cholesterol clefts show T1-weighted high- and T2-weighted low-signal intensities, that hemosiderin cysts containing xanthogranuloma-like fluid show T1-weighted high/iso- and T2-weighted high-signal intensities and that thick fibrosis (granulation) show both T1-weighted and T2-weighted low-signal intensities [14]. The mixed signal intensities on both T1- and T2-weighted images with heterogeneous enhancement reflect their complex histologic components. The cholesterol deposits of a xanthogranuloma appear hyperintense on T1-weighted images; however, calcifications, micro- or focal hemorrhages and hemosiderin deposits contained in tumors can frequently result in heterogeneous cystic lesions and may be essential in the formation of xanthogranulomas as the trigger for granulomatous changes or as a result of these changes.

A MEDLINE English-language literature search commencing from 2000 revealed eight separate reports of cases of sellar xanthogranuloma [4-7,9,15]. MRI was performed in all eight of the reviewed cases (Table 3). Eight previous cases of sellar xanthogranuloma with MRI findings manifested hypo- and heterogeneous intensity on T2-weighted imaging studies. For seven of nine reported cases (including the present case), mixed high-intensity lesions were seen on T1-weighted imaging and, generally, low or heterogeneous intensity on T2-weighted imaging.

**Table 3:** Reviewed cases of xanthogranuloma^a^

Case	Author	Age/sex	Symptoms	Clinical findings	Radiological findings (MRI)	Tumor size (cm)	Time to operation	Tumor region	Follow-up
1	Present case	47/M	Headache Body weight loss	Panhypopituitarism Visual disturbance	T1:H/I, T2:CE:heterogeneous Mixed-intensity solid tumor	1.2 × 1.8 × 1.5	5 M	Intrasellar	Required medical supplement
2	Reithmeier *et al*., 2002 [4]	51/M	Pallor Loss of libido	Hypophyseal insufficiency Visual dysfunction	T1:H, CE:heterogeneous	Unavailable	Unavailable	Intrasellar	Not described
3	Yonezawa *et al*., 2003 [5]	67/M	Headache General fatigue Appetite loss	Panhypopituitarism	T1:H, T2:L, heterogeneous:CE: N Mixed-intensity cystic tumor	Not described	3 M	Intrasellar	Doing well 3 months later
4	Burt *et al*., 2003 [6]	29/M	Headache Nausea Erectile dysfunction	Hypophyseal dysfunction Visual defects	T1:H, T2:heterogeneous CE:heterogeneous Mixed-intensity partially cystic tumor	1.8 × 1.5	3 W to 3 M	Suprasellar	Doing well 18 months later
5		26/M	Body weight loss Lethargy	Panhypopituitarism	T1:H, T2:L, CE:N Mixed-intensity cystic tumor	Not described	12 M	Intrasellar-suprasellar	Required medical supplement
6	Jung *et al*., 2005 [9]	57/F	Headache	Visual disturbance	T1:H, T2:L, CE:heterogeneous Mixed-intensity solid tumor	2.0 × 2.0 × 2.5	12 M	Intrasellar-suprasellar	Not described
7		5/M	Weakness Appetite loss Headache	Hypothyroidism Secondary adrenal insufficiency Diabetes insipidus	T1, T2:heterogeneous Mixed-intensity cystic tumor	2.6	5 W	Intrasellar-suprasellar	Not described
8	Liu et al., 2008 [22]	32/M	Consciousness disorder	Blurred vision Disorientation	T1:H, T2:H and focal L Mixed-intensity cystic tumor	3.4 × 3.8 × 4.2	2 D	Suprasellar	Doing well 6 months later
9	Sugata *et al*., 2009 [7]	26/M	Polyuria General fatigue	Hypopituitarism Visual disturbance	T1:I or L, T2:L, CE:heterogeneous Mixed-intensity solid tumor	3.0	5 to 10 Y	Suprasellar	Required medical supplement

^a^H, high intensity; L, low intensity; I, isointensity; heterogeneous, heterogeneous intensity; CE, gadolinium contrast enhancement; N, negative; Y, years; M, months; D, days; T1, T1-weighted; T2, T2-weighted.

A xanthogranuloma of the sellar region usually causes symptoms of an endocrine nature, despite favorable survival outcomes for patients with xanthogranulomas. In this case, a good outcome is defined as a socially independent life without impairment but no recovery of endocrine function. The appropriate therapeutic strategy for most cases of xanthogranuloma of the sellar region appears to be to correct the underlying endocrine problems first. Patients often need to receive long-term endocrine replacement therapy to improve their symptoms. In the eight cases of xanthogranuloma in the literature that we reviewed, no examples of recurring xanthogranuloma of the sellar region were noted, and the overall outcome for patients with this condition appeared to be favorable (Table 3). However, only three of eight patients whose intrasellar tumors were removed within 3 months of clinical onset recovered without requiring endocrine replacement therapy (Table 3). Another three patients, including the patient described in our present case report, required medical support because of mild hypopituitarism after the operation. The outcomes of the other three patients were not described (Table 3). Even if the tumor size is small, an intrasellar tumor or delayed surgical intervention probably results in unfavorable endocrine outcomes. On the contrary, despite the presence of tumors 3 cm or larger in diameter, suprasellar lesions appear to carry a better endocrinological prognosis if appropriate treatment is initiated as soon as possible. The increased risk of permanent pituitary dysfunction in various sellar lesions undergoing xanthogranulomatous reaction is probably related to their cause: hemorrhage, inflammation or degeneration. Therefore, surgical intervention early in the development of these changes may improve pituitary function [5,6,15]. This seems to suggest that surgical intervention within 3 months of clinical onset and the tumor's being in a non-intrasellar location are important factors that result in favorable postoperative pituitary function. It is possible that early surgical decompression resulted in recovery of pituitary function in cases 3, 4 and 8, but that chronic inflammation in cases 1, 5 and 9 resulted in destruction and fibrosis of the pituitary as well as irreversible hormonal deficits (Table 3).

## Conclusion

Because xanthogranulomas of the sellar region are rare, their nature and clinical course remain somewhat unclear. The seriousness of the hypopituitarism, the hyperintensity of T1-weighted MRI or hypointensity of T2-weighted MRI, and the absence of calcification should alert clinicians to the possibility that the tumor may be a xanthogranuloma. Therefore, xanthogranulomas should be included in the preoperative differential diagnosis of intrasellar tumors. Moreover, we suggest that such tumors should be treated as soon as possible, within a minimum of 3 to 4 months, especially in the case of tumors occupying the intrasellar region, to improve endocrinological outcomes, even if the tumors seem small.

## Consent

Written informed consent was obtained from the patient for publication of this case report and any accompanying images. A copy of the written consent is available for review by the Editor-in-Chief of this journal.

## Competing interests

The authors declare that they have no competing interests.

## Authors' contributions

TN and KM was major contributor to the writing of the manuscript. TN, KM, HI and TI analyzed and interpreted the patient data regarding endocrinological disease. YK and RH performed the histological examination of the xanthogranuloma. NK and TT performed neurosurgery on the patient. All authors read and approved the final manuscript.
